# Investigating Changes in Social Networks Following Conflict in Zoo‐Housed Bonobos (*Pan paniscus*)

**DOI:** 10.1002/ajp.70047

**Published:** 2025-05-26

**Authors:** Sedona Epstein, Mariam Fischer, Sara Cotton, Frances White

**Affiliations:** ^1^ Department of Anthropology University of Oregon Eugene OR USA

**Keywords:** affiliation, aggression, proximity, reconciliation, sociosexual behavior

## Abstract

Bonobos (*Pan paniscus*) are successful at managing conflict and promoting group affiliation, but it is still uncertain how conflict affects the entire social group, particularly in captive populations. We conducted social network analyses using dyadic proximity data to understand the structure of five alternating social group compositions of a population of zoo‐housed bonobos, using measures of centrality and social strength. We then compared social network measures between neutral, post‐conflict (intergroup and intragroup), and post‐single‐party outburst (scream, display to the public) contexts to determine how conflict influences social behavior within the entire social network. We found that, across conflict contexts, dominant females have higher social group centralities than other individuals. Dominant females also received social initiations at a higher rate than others, suggesting this centrality is structurally maintained by other individuals. Further, rates of proximity are significantly higher following single‐party outbursts than in other contexts, which indicates an important social distinction between this context and others and may be best explained by considering these outbursts as signals of group‐level social tension, during which group members may seek social interaction and reassurance. Consistent differences in proximity rates were observed between dominant females and other group members, indicating that the social role of the dominant female in a bonobo social group across conflict contexts is different from that of other bonobos. The results of this study emphasize the flexible nature of bonobo sociality, highlight the distinct and important role of dominant females within the social network, and present social network analysis as a useful methodological tool for studying group‐level changes in bonobo sociality and behavior.

## Summary

- Dominant female bonobos had higher social group centralities and received proximity initiations at a higher rate than others.
- Rates of proximity changed across conflict contexts and were highest following single‐individual outbursts, indicating that the entire group displays social and behavioral flexibility following instances of conflict and unrest.
- Social Network Analysis is a recent and useful tool to effectively study group‐level change in primate social structures and behavior.

## Graphical abstract

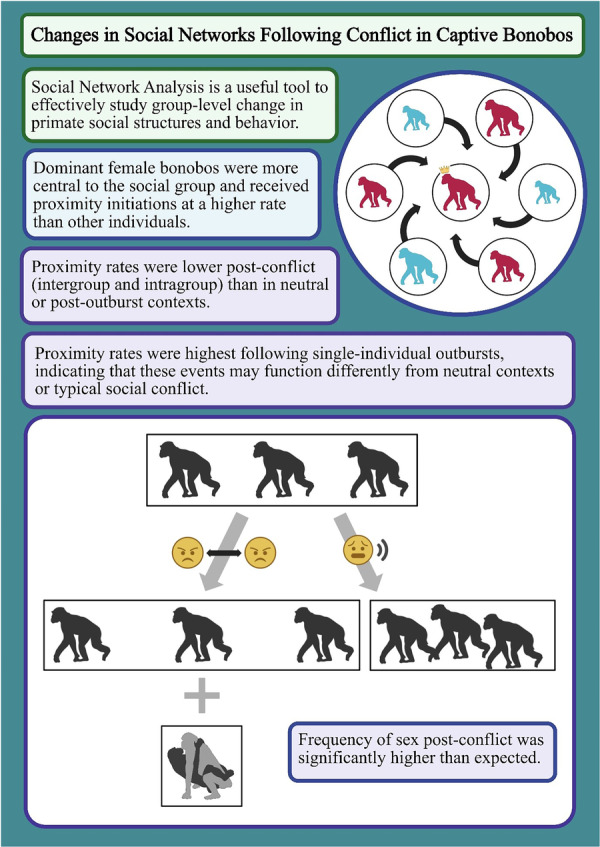

**Abbreviations**
CCT: conciliatory tendency
MCZ: milwaukee county zoo
PC‐MC: post conflict‐matched control
SNA: social network analysis

## 1 Introduction

Researchers investigating the evolution of human conflict mitigation, resolution, and prevention often turn to one of our closest relatives: bonobos (*Pan paniscus*). Bonobos have been shown to exhibit conflict‐related behaviors that work to reduce frequency and intensity of conflict, including sociosexual behaviors, strong female‐female bonds and coalitions, conflict interventions, reconciliations, and consolations (Kano 1992; White 1996; White 1989; White and Thompson‐Handler 1989; Vervafcke et al. 2000; Palagi et al. 2004; Clay and de Waal 2013; Clay and de Waal 2015; Gruber and Clay 2016; Boose and White in review; Samuni and Surbeck 2023; Ryu 2024).

Bonobos show female dominance dependent on strong social bonds between unrelated females (White and Wood 2007). This coalition‐driven female dominance results in flexible dominance hierarchies that depend on group composition, which naturally varies under their fission‐fusion social system (White and Lanjouw 1992; Gruber and Clay 2016). Compared to their male‐dominant closest relatives, chimpanzees, bonobos show far more intergroup tolerance and far less severe or lethal conflict, sexual coercion, and proactive aggression (Furuichi 1989; White and Wood 2007; Paoli 2009; Wrangham 2018; Gruber and Clay 2016; Samuni and Surbeck 2023). These behaviors and general characteristics of bonobo social systems are what lend bonobos a reputation for being a ‘peaceful primate’ (White et al. 2013), and though there are many needed nuances to this moniker (Mouginot et al. 2024), bonobo conflict‐related social behaviors and strategies may be key to understanding how humans have evolved our own successful conflict‐related social strategies (Jaeggi et al. 2016; Samuni et al. 2024). Whereas reconciliation and conflict‐related behavioral changes have been studied in individuals directly involved in conflicts, there are few group‐level studies in bonobo social connectedness surrounding conflict, particularly while also considering the role of dominant females in a bonobo social group. A greater understanding of the role of female dominance and of group‐level behavioral changes that occur in bonobo social groups throughout conflict is needed to properly contextualize dynamics surrounding conflict, reconciliation, and social peace in bonobos.

This study uses social network analysis (SNA) (Wasserman and Faust 1994; Freeman 2004; Wey et al. 2008; Kasper and Voelkl 2009) on a population of bonobos at the Milwaukee County Zoo (MCZ) to (1) determine if dominant female bonobos are most central to affiliative social networks, and if so, (2) determine if this centrality is maintained by social initiations from dominant females to others or from others towards dominant females, (3) assess changes in rates of dyadic proximity of dominant females and of Nondominant individuals across conflict contexts, and (4) compare rates of sociosexual encounters across conflict contexts.

Social network analysis is a useful methodology that can assess social dynamics of an entire social group, taking into account direct connections (number and strength of connections) and indirect connections (connectedness of connections), both of which are important to an individual's social relationships and overall standing within a group (Kasper and Voelkl 2009; Sueur et al. 2011; Ellis et al. 2019). The SNA statistics we focus on in this study are eigenvector centrality and strength. Though there are other SNA‐produced measures of centrality, eigenvector centrality is an indirect measure of an individual's embeddedness in a social network, making it an apt statistic to understand which individuals are the most centrally connected and important in a group of highly social primates. Strength is a direct measure of the total frequency of behaviors measured. In a directed network, which considers the directionality of a social behavior (‘A initiates grooming with B’ instead of ‘A and B groom’), strength can be divided into ‘in‐strength’ and ‘out‐strength.’ In‐strength measures the total frequency of behaviors an individual received, whereas out‐strength measures the total frequency of behaviors an individual initiated (Torfs et al. 2023). Measures of strength can be useful in comparing dyadic relationships as well as comparing sociality, tendency to initiate, and tendency to receive social behaviors between individuals. Compared to other species, there are not many SNA studies that have been conducted on bonobos, so clear dynamics or roles based on sex, age, and dominance that operate through these SNA measures are continuing to be understood and contextualized.

Bonobo dominance hierarchies influence social connectedness and centrality; Torfs et al. (2023) applied SNA to 22 groups of zoo‐living bonobos and found that, for females, both eigenvector centrality and dominance showed a quadratic relationship with age, with highest values of both in middle‐aged females, whereas, for males, eigenvector centrality was not significantly tied to age and dominance was highest in younger males and lowest at around younger middle age. They did not report if female dominance was correlated with eigenvector centrality, though these results certainly warrant investigating this possibility. That bonobos are generally female‐dominant (especially under zoo‐living conditions, where change in group compositions is limited) further argues for a possible link between female dominance and group centrality (Vervaecke et al. 1999). Tokuyama and Furuichi (2017) reported that eigenvector centrality from grooming networks positively correlated with frequencies of initiation of collective movements, and that older females had high initiation indexes. Further, high‐ranking female bonobos are more attractive grooming partners than other individuals (Franz 1999), and grooming rates among primates typically also correlate with rates of other social behaviors, such as proximity and co‐feeding (Ventura et al. 2006; King et al. 2011; Silk et al. 2013; Samuni et al. 2018; Staes et al. 2022). Torfs et al. (2023) also reported that, like female dominance, out‐strength (among females) and in‐strength (both sexes) were lowest in young and old bonobos.

Social network analysis can also provide clarity on how social groups may change over time or under different conditions. There are currently no SNA studies on how bonobo social groups change across disruptive events, though previous studies using different methodologies have established clear evidence of post‐conflict reconciliation in bonobos, often through sociosexual contact. de Waal (1987) reported increased rates of affiliative and sociosexual behaviors immediately following conflict. Hohmann and Fruth (2000) reported increased rates of g‐g rubbing in the 15 min following conflict compared to the 15 min before conflict. Most studies on reconciliation and consolation use the standard Post Conflict‐Matched Control (PC‐MC) method, in which the observer does a 10‐min focal of the victim immediately following conflict and compares it to a neutral 10‐min focal from the same time on the preceding day. The timing of the first affiliative contact between former opponents post‐conflict is compared to the timing of the first affiliative contact in a matched control period. If the post‐conflict dyad engages in affiliative contact before the matched control, this is considered reconciliation (de Waal and Yoshihara 1983; Wittig and Boesch 2005; Roselyn 2015; Clay and de Waal 2015). Palagi et al. (2004) assessed conciliatory tendencies (CCT) in captive bonobos, defined as the number of pairs that reconciled minus the number of pairs that did not, divided by the total number of PC‐MC pairs (Veenema et al. 1994), and found a mean individual CCT = 35.6%, though this number was higher among female dyads and dyads with greater overall relationship quality. Reconciliation in this Palagi et al. study appeared as both affiliative behaviors and sociosexual behaviors. Fortunato (2009), also assessing CCT in captive groups, found a mean individual CCT = 40%, but with no correlation to sex or relationship quality. Roselyn (2015) found, among San Diego Zoo bonobos, a mean individual CCT = 10.11%, with significantly lower rates of sex than expected during reconciliation. These studies vary in their reported rates of CCT and general frequencies of affinitive behaviors versus sociosexual behaviors, which could be due to a number of factors, including enclosure size/space available, individual history and personality, group compositions, and captive care protocols. Regardless, these studies do show a clear presence of post‐conflict affiliation, and variation in results may demonstrate bonobo behavioral flexibility and variation.

Restricting observation times to the initial 10 min following conflict, as in this PC‐MC method, is particularly useful in attributing post‐conflict behaviors of individuals directly to the preceding conflict and focusing on those individuals involved in said conflict. However, SNA requires a longer time frame to collect sufficient data (particularly with one observer at a time, such as in the current study). Further, we are interested in how the connectedness of the entire social group may change following conflict, not just behaviors involving the conflict victim. Bonobos are highly socially intelligent and flexible, with a high propensity for understanding group dynamics and hierarchy changes that often lead to changes in group composition. This makes it likely that the entire social group, not just those involved in a conflict, may adjust their social behaviors following (and possibly preceding) conflict (Vervaecke et al. 1999; Gruber and Clay 2016). Applying SNA methodology to the study of reconciliation and other post‐conflict affiliations will broaden both our focus (the whole social group) and the time frame of post‐conflict analysis.

Based on our understanding of bonobo female dominance and reconciliation behaviors, we predict that (1) dominant females will have higher eigenvector centralities than other individuals, (2) dominant females will have higher in‐strength and out‐strength rates than other individuals, (3) total strength rates of both dominant females and Nondominant individuals will differ across conflict contexts, with higher rates post‐conflict, and (4) rates of sociosexual encounters will be higher following conflict contexts than neutral or outburst contexts.

## 2 Methods

### 2.1 Ethical Statement

All research reported in this study was observational and noninvasive. This study also complies with all standards set forth by the University of Oregon Institutional Animal Care and Use Committee (Proposal: IPROTO202300000013, Approved May 22, 2023) and adheres to the American Society of Primatologists (ASP) Principles for the Ethical Treatment of Nonhuman Primates.

### 2.2 Study Area and Subjects

All observations were conducted at the Milwaukee County Zoo (MCZ), in Milwaukee, Wisconsin, from public viewing areas. On‐exhibit enclosures include a 75,000 ft^3^ indoor exhibit and a smaller outdoor exhibit consisting of many connected metal mesh tunnels (Drews et al. 2011; Orr 2014). The MCZ houses 18 bonobos (Table 1), which were in primarily continuous full contact within artificially constructed and alternating groups in two outdoor enclosures, one indoor enclosure, or off exhibit. Using age classifications from Boose and White (2017), this population consisted of seven adult females (ages 13–27), four adult males (ages 25–34), three adolescent females (ages 9 and 10), three subadult males (ages 8, 9, and 12), and one juvenile male (age 7). At the request of the MCZ, we replaced bonobo names with ID codes when reporting data. MCZ primate zookeepers rearranged group compositions every 1–4 weeks, keeping family groups (mother plus offspring) together and individuals prone to conflict apart (zookeepers, personal communication). Individual M2, an adult male, was grouped with his sister's (F3) family group due to a large social change 7 months prior, in which their mother and dominant female died. F3 then became dominant, while M2's dominance plummeted, so M2 was grouped with his sister for protection and affiliation (zookeepers, personal communication).

**Table 1:** Milwaukee County Zoo bonobos.

ID	Sex	Age class	Age	Mother
F1	F	Adult	27	
F2	F	Adult	24	
F3	F	Adult	20	Deceased Former Dominant Female
F4	F	Adult	20	
F5	F	Adult	19	
F6	F	Adult	18	
F7	F	Adult	13	F2
F8	F	Adolescent	10	
F9	F	Adolescent	10	
F10	F	Adolescent	9	F2
M1	M	Adult	34	
M2	M	Adult	33	Deceased Former Dominant Female
M3	M	Adult	28	
M4	M	Adult	25	
M5	M	Subadult	12	F3
M6	M	Subadult	9	F4
M7	M	Subadult	8	F5
M8	M	Juvenile	7	F3

Though changing group compositions are a feature of wild bonobo social systems, there are important differences between the social dynamics of the MCZ bonobos and wild fission‐fusion: the MCZ bonobos never fully fuse or fission but alternate between groups of around 3–7 individuals selected by zookeepers. Typically on a weekly basis, zookeepers rearrange group composition out of public view, without fusion or fission to keep certain dyads that are prone to fighting apart (zookeepers, personal communication). This management meant that multiple individuals were never together in the same group during this study, so all individuals cannot be combined into a single network. Therefore, social networks were constructed based on each group as observed. Because groups were frequently rearranged, some individuals were present in two groups, making it possible to observe behavioral changes, including dominance, based on group composition. Although all individuals are familiar with each other in some capacity (visual/olfactory/auditory if permanently kept apart), the lack of fusion in this population means that we consider compositions of individuals within the same enclosure on any given day to form artificial groups, not parties, though we acknowledge that intergroup relationships in this population likely differ from wild groups, who do not frequently recombine compositions. Over the observation period, five groups of distinct compositions were recorded and observed (Table 2). Six individuals appeared in two groups, and twelve individuals appeared in only one group. Age, recent group histories, and genetic relation was determined from personal communication with zookeepers and online resources (Ape Cognition and Conservation Initiative © 2024).

**Table 2:** Group compositions and minutes of focal data collected per conflict context and individual, by group. Lightly shaded cells indicate that the category was excluded from centrality analyses and darker shaded cells indicate that the individual within the category was also excluded from strength analyses. Groups A, B, and D had no access to another group, so intergroup encounters were not possible. We observed no single‐individual outbursts in group E. Every intergroup conflict was between groups C and D. However, observations for these groups were never conducted simultaneously, so all conflicts recorded were separate instances. Some group categories included smaller amounts of focal data than others and therefore must be interpreted with caution.

Focal data collected by group and conflict categories								
Group	Individuals	Minutes observed via focal per conflict context				Dominant female	Conflicts observed (Count)	Location
		Neutral	Outburst	Intragroup	Intergroup			
A	F3	33	10	10		F3	Intragroup (2)	Indoor enclosure
	M5	32	8	10			Outburst (3)	
	M8	23	10	10				
	M2	30	7	20				
	F5	26	10	20				
	M7	29	0	10				
B	F3	174	30	4		F3	Intragroup (5)	Indoor enclosure
	M5	125	49	20			Outburst (8)	
	M8	139	25	23				
	M2	152	30	9				
	F2	184	30	0				
	F7	137	33	0				
	F10	130	43	5				
C	F5	65	10	2	45	F5	Intragroup (2)	Outdoor enclosure (Access to Group D through mesh)
	M7	30	10	2	75		Outburst (1)	
	M1	36	10	20	50		Intergroup (9)	
	F9	50	10	10	50			
D	F4	33	10	0	46	F4	Intragroup (3)	Outdoor enclosure (Access to Group C through mesh)
	M6	20	21	10	30		Outburst (3)	
	F6	17	30	7	37		Intergroup (5)	
	M3	20	25	10	20			
E	F1	74		0		F1	Intragroup (1)	Indoor enclosure
	F8	66		3				
	M4	70		10				

### 2.3 Data Collection

In the data collection process, we noted social state behaviors continuously (grooming, play, cofeeding, contact, close proximity, partial proximity) (Stevens et al. 2023), recording the most inclusive behavioral category (see Table 3 in supplement for ethogram). For example, grooming includes contact but is recorded as grooming. Grooming, play, and cofeeding did not occur at the same time. However, there was not enough data on each behavior alone (e.g., grooming) to conduct single‐behavior SNAs. For example, our largest data set, Group B, captured only 10 dyads grooming out of 42 dyads that were observed to be social in this group. Further, F3 groomed M2 at a far higher rate than any other dyad in the group (20/72 min of total grooming observed), which biases SNA statistics and inflates M2's scores, as he was otherwise not very social. Therefore, we pooled all affiliative social state behaviors that required proximity (within 2 meters) into a single category. Proximity has been established as valid indicator for bonobo and chimpanzee social bonds and affiliation and is a convenient and valid behavior to use when creating primate social networks (White and Burgman 1990; White and Chapman 1994; Parish 1996; Mitani 2009; Kasper and Voelkl 2009; Moscovice et al. 2019; Torfs et al. 2021; Yokoyama and Furuichi 2023). We used nonparametric Spearman Correlations to examine if the total affiliative behaviors recorded (grooming, cofeeding, playing) were correlated with the total proximity measures recorded (contact, close proximity, partial proximity).

We collected behavioral data between July and September of 2023, through 10‐min focal observations using ZooMonitor (Ross et al. 2016). One complete session was 10‐min focals of everyone present in the group. Because groups were not equal in numbers, sessions varied in length. Sessions were recorded during MCZ public hours, which typically fell between the hours of 9:00 am and 3:00 pm. We obtained a total of 59 sessions. For all social state behaviors, we noted duration (sec) and who initiated and terminated the interaction when observed (ex: individuals who present themselves to be groomed or individuals beginning grooming with no prompting are initiators). Sociosexual behaviors, which typically last no more than a few seconds, were recorded as counts and, therefore, were analyzed separately from durational behaviors. Sociosexual behaviors did not occur frequently enough to create a social network in each group, but descriptive statistics are reported here.

We recorded all occurrences of aggression, submission, and sociosexual interaction from all group members (Stevens et al. 2023). We defined a conflict as an interaction between two or more individuals that included aggressive behavior, submissive behavior, or both. When a conflict occurred, we recorded the conflict data and then continued with the same focal from before the conflict. Conflicts between individuals in the same group were considered intragroup conflicts. Conflicts between individuals in different groups were considered intergroup conflicts. Due to the exhibit structure, only groups C and D had any exposure to another group, and therefore, only these groups could engage in intergroup conflict. Typical intergroup conflicts involved multiple individuals from both groups vocalizing, charging, displaying, and striking or pushing objects against the barrier between the groups. Whereas intragroup conflicts could be similar in behavior and intensity, the aggressive behaviors observed were usually less intense, and the submissive behaviors were typically more overt. Occasionally, an individual would exhibit aggressive or submissive behavior with no apparent recipient, most commonly hitting the enclosure glass/mesh or screaming with no discernable trigger or target. We categorized these behaviors as single‐individual outbursts. To consider behaviors that occur after 10 min post‐conflict and to gather enough data on the entire social group for social network analyses, we extended our definition of ‘post‐conflict’ to 1 h after a conflict has occurred. The hour following an intragroup conflict was considered post‐intragroup, the hour following an intergroup conflict was considered post‐intergroup, the hour following a single individual outburst was considered post‐outburst, and when an observation did not have a conflict or outburst in the hour preceding it, it was considered neutral.

### 2.4 Data Analysis

We conducted social network analyses (SNA) on each social group, for each category: neutral, post‐intragroup, post‐intergroup, and post‐outburst. The data for these SNAs are total seconds within proximity. We used directed SNAs, with the initiator of a social interaction bout as the source and the recipient of a social interaction bout as the target. This means that dyads are represented directionally (ex: F3 [source] →F5 [target] AND F5 [source] → F3 [target]). We calculated the total number of seconds each directed dyad was within proximity. To control for observation time within a network, we divided this number by the total seconds each dyad member was observed to produce the dyadic ‘weight’ input for SNA. For example, in group A's neutral context, F3 was observed for 33 min and F5 was observed for 26 min, and they were in proximity (when initiated by F3) for 171 s, so the dyadic weight for (Group A, Neutral) F3 [source] → F5 [target] was 171/59 = 2.898. The formula used is as follows:

$$\mathrm{Dyadic Rate of}\;A\to B=\frac{\mathrm{Seconds dyad engaged in behavior}}{\left(\mathrm{Minutes observing}\;A\right)+\left(\mathrm{Minutes observing}\;B\right)}$$

We ran 14 social network analyses and produced individual‐level scores for eigenvector centrality, in‐strength, out‐strength, and total strength. Eigenvector centrality was calculated to standardize between different group sizes, producing a statistic scaled from 0 to 1, with 1 being the most centralized individual. In‐strength is calculated as the sum of rates in which an individual receives initiations from others. Out‐strength is calculated as the sum of rates in which an individual acts as an initiator. Total strength is the sum of in‐strength and out‐strength and is a measure of overall sociality. When there is an effect of group size on network measure, it is common to standardize for group size (eg: Torfs et al. 2023). Using nonparametric Spearman Correlations, we tested for a group size effect on in‐strength, out‐strength, total strength, and centrality to determine if a group size correction was needed.

To check for normality, we used a Kolmogorov‐Smirnov test on centrality, in‐strength, and out‐strength by dominant female status and for total strength by conflict state. In‐strength, out‐strength, and total‐strength were not normal and could not be transformed to normality, so we used non‐parametric tests for these categories by converting each strength measure to ranks (Sokal and Rohlf 1987). To assess the validity of classifying individuals into categories of ‘Dominant Females’ and ‘Other Individuals’, we used ANOVAs to compare centrality, in‐strength, out‐strength, and total strength between individuals for each category. As individuals were not significantly different from each other, individuals within categories could be combined for further analyses.

Dominance was determined by dyadic displacement rates and confirmed by zookeeper communication. Dominance status was divided into two categories: dominant female (the most dominant female in any given group) and Nondominant individual (all other individuals, including females that were not the most dominant in the group and all males; also referred to as “others”). The most dominant female in the population was F3, so she was the dominant female in any group she was placed in. Notably, the dominant female in group C, F5, was also present in group A, but was not the dominant female when (the higher‐ranking) F3 was present. Therefore, F5's dominance status was group composition dependent. Flexible and compositionally dependent dominance is common in bonobos (Gruber and Clay 2016), but because of the restricted nature of zoo‐controlled group composition changes, and because we labeled only the most dominant female, no other individuals changed dominance status between groups.

To determine if dominant females differed in centrality to other individuals, we compared eigenvector centrality scores from the 14 networks between dominant females and others using an ANOVA (Sokal and Rohlf 1987). To determine if dyads involving dominant females differ in directionality rates from other dyads, and how these rates might change across conflict contexts, we used two‐way ANOVAs for in‐strength and out‐strength using dominance and conflict context as main effects and testing for significant interactions (Sokal and Rohlf 1987). To compare overall rates of social behaviors across conflict contexts, we used a non‐parametric ANOVA with a priori, orthogonal multiple comparisons on ranked total strengths to identify significant differences among different conflict contexts, comparing post‐conflict vs non‐conflict, post‐intragroup vs post‐intergroup, and neutral vs post‐single individual outburst (Sokal and Rohlf 1987). Finally, to compare the frequency of sociosexual behaviors between conflict and non‐conflict contexts, we used a *log likelihood*, replicated goodness of fit G‐test (Sokal and Rohlf 1987), using observation time as the expected frequency.

In an SNA data set, if two or more individuals were not observed via focal sampling, then dyadic interactions between them would be impossible to observe, making the resulting centrality scores within the group inaccurate, as the network data would be incomplete. Group B's post‐intragroup category did not include any focal data for two individuals, so we were unable to witness any social interactions between them, thus affecting their social network outputs. Therefore, strength scores from these two individuals and all centrality scores from Intragroup B were excluded from analyses. Further, there was not enough dyadic information post‐intragroup for groups D and E to produce centrality scores, although there was enough data for these groups to be included in post‐intragroup strength analyses. Focal observation time varied between individuals and groups, which is a limitation of this study. We report the distribution of focal observations (Table 2) and interpret our analyses with the information and acknowledgement that some categories are based on fewer minutes of observation and therefore must be interpreted with caution.

## 3 Results

Spearman Correlations showed that affiliative and proximity data were correlated (r = 0.21973, *p* = 0.0397). It is possible that different affiliative behaviors represent different dyadic relationships and meanings, but given this degree of correlation, they are all represent some form of dyadic cohesion.

### 3.1 Dominant Female Centrality

**Figure 1:**
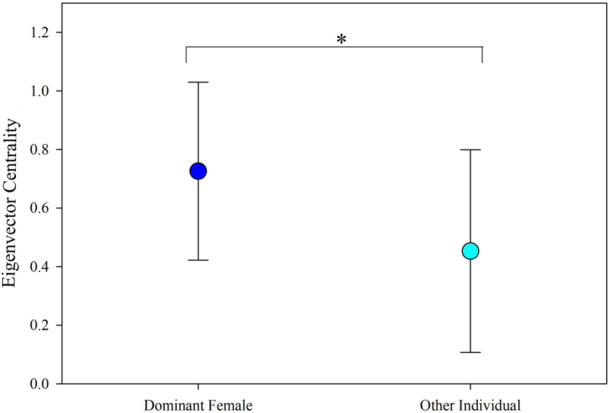
Average eigenvector centrality differences between dominant females and other individuals (nondominant females and all males) with standard deviations: Dominant females show significantly higher centrality scores in social networks based on affiliative social state behavior.

**Figure 2:**
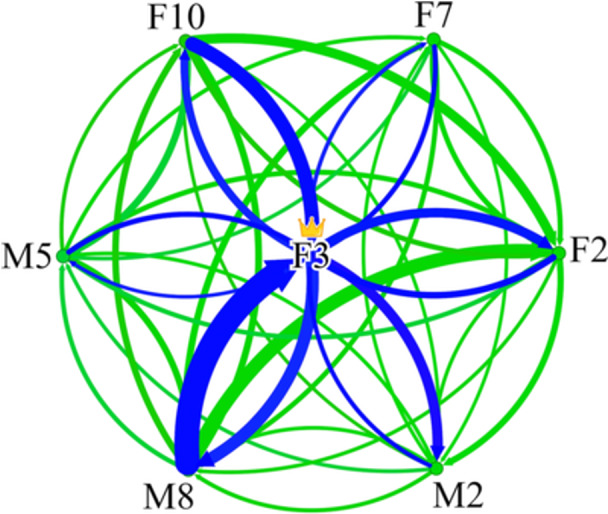
Social network example of dominant female centrality: Group B (all conflict contexts combined). Arrows represent initiations between dyads and lines are weighted by directed dyadic strength (seconds within proximity). To show the centrality of F3, the dominant female, connections involving her are blue and all other connections are green.

Distribution of centrality scores was not significantly different from a normal distribution within both dominant females (Kolmogorov‐Smirnov, D = 0.201052, *p* > 0.15) and other individuals (Kolmogorov‐Smirnov, D = 0.111584, *p* = 0.1201). Centrality scores also did not significantly vary among dominant females (F = 0.84, *df* = 3, 9, *p* = 0.5051) or among other individuals (F = 1.38, *df* = 14, 35, *p* = 0.2154). As predicted, dominant females had significantly higher eigenvector centrality scores than other group members (F = 6.73, *df* = 1, 61, *p* = 0.0119) (Figure 1). This indicates that dominant females were more central to their groups than other individuals. See Figure 2 for an example of a social network (Group B) highlighted to emphasize dominant female centrality.

### 3.2 Dominant Female In‐Strength and Out‐Strength

The Spearman Correlations for possible group size effects showed that group size and SNA measures (in‐strength, out‐strength, total strength, and centrality) were not correlated (In‐Strength: *r* = 0.13203, *p* = 0.2588; Out‐Strength: *r* = 0.08219, *p* = 0.4833; Total Strength: *r* = 0.10524, *p* = 0.3689; Centrality: *r* = −0.18094, *p* = 0.1559), so no correction factor was needed.

Distributions of in‐strength and out‐strength were significantly different to normal within some conflict states among Nondominant individuals (In‐Strength: Dominant Female = Y: Neutral D = 0.22396, *p* > 0.1500, Outburst D = 0.240037, *p* > 0.15, Intragroup D = 0.326587, *p* = 0.0788, Intergroup D = 0.26025, *p* > 0.15; Dominant Female = N: Neutral D = 0.189314, *p* = 0.0729, Outburst D = 0.235208, *p* = 0.0136, Intragroup D = 0.256508, *p* < 0.01, Intergroup D = 0.261603, *p* > 0.15; Out‐Strength: Dominant Female = Y: Neutral D = 0.19623, *p* > 0.15, Outburst D = 0.253504, *p* > 0.15, Intragroup D = 0.331998, *p* = 0.0696, Intergroup D = 0.26025, *p* > 0.15; Dominant Female = N: Neutral D = 0.268343, *p* < 0.01, Outburst D = 0.198387, *p* = 0.0751, Intragroup D = 0.409453, *p* < 0.01, Intergroup D = 0.218964, *p* > 0.15), so we converted in‐strength and out‐strength variables to ranks for non‐parametric comparative analyses. Ranked rates were also used for assessing categorical variability for Nondominant individuals, though ranked transformation was not needed for assessing categorical variability among dominant females. Among dominant females, in‐strength and out‐strength rates did not vary significantly (In‐strength: F = 1.74, *df* = 3, 12, *p* = 0.2126; Out‐strength: F = 1.05, *df* = 3, 12, *p* = 0.4049) Among Nondominant individuals, ranked in‐strength rates did not vary significantly (F = 1.24, *df* = 14, 44, *p* = 0.2849), though ranked out‐strength rates did vary significantly (F = 2.66, *df* = 14, 44, *p* = 0.0067). We therefore assessed Nondominant categorical variability in ranked out‐strengths by conflict context, as we expected (and found) that conflict context would influence these rates. Nondominant individuals did not significantly vary in their ranked out‐strengths by conflict context (Neutral: F = 2.04, df = 14, 4, *p* = 0.2569; Single: F = 1.77, *df* = 12, 4, *p* = 0.3074; Intragroup: F = 3.32, *df* = 12, 4, *p* = 1.283), though there was not enough data to conduct this assessment within the intergroup context. Generally, these results show that our categories of ‘dominant females’ and ‘others/nondominant individuals’ are valid for the purposes of our analyses, though it will be beneficial for future research to investigate possible sub‐categories within ‘nondominant individuals’ that may influence social behavior and network results.

**Figure 3:**
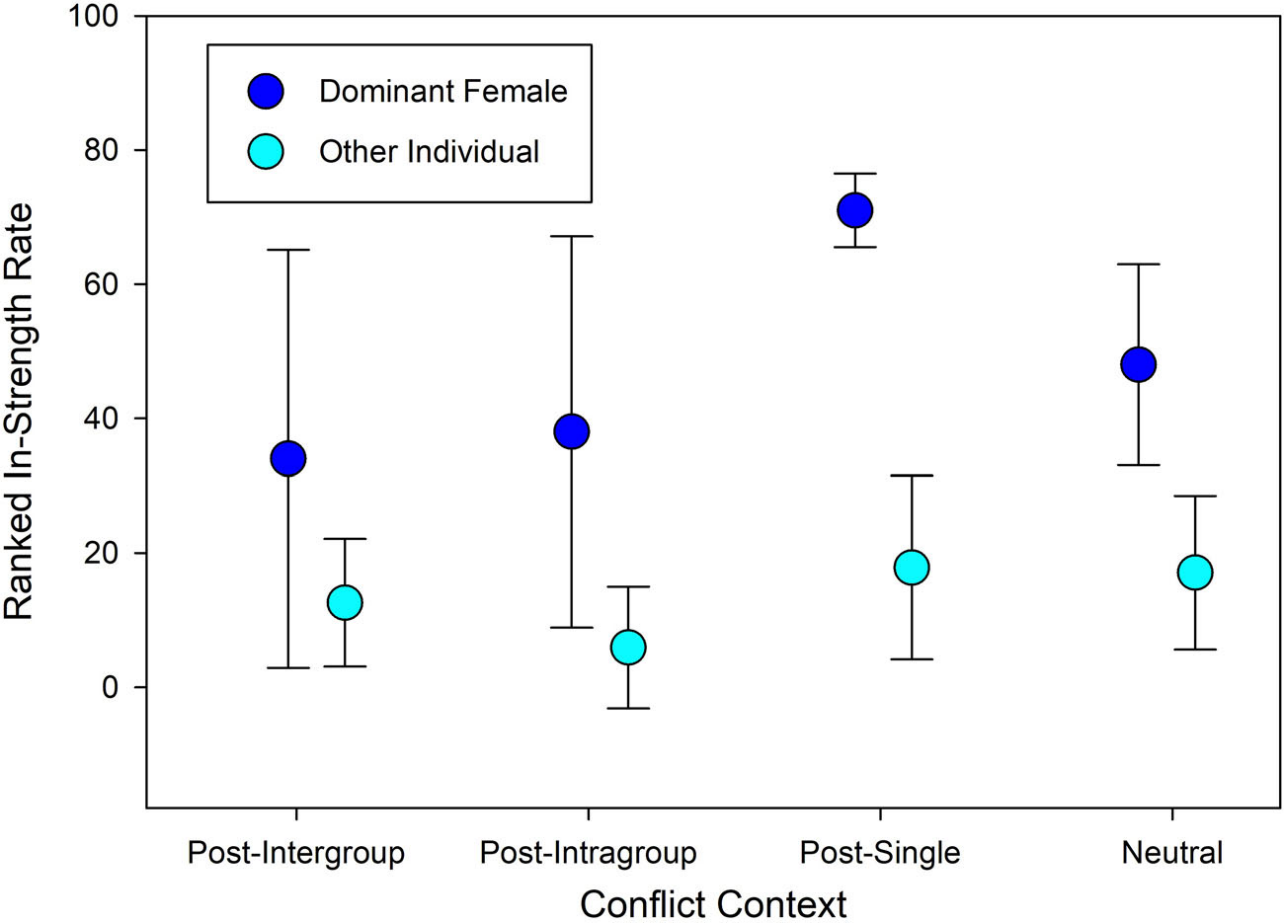
Ranked in‐strength rate comparisons between dominant females and other individuals across four conflict contexts with standard deviations: In‐strength rate is measured as the rate of time spent in dyadic proximity bouts initiated by another individual. These rates are converted to ranks for a non‐parametric ANOVA. Dominant females show higher in‐strength rates overall, with rates varying between conflict contexts. For both dominant females and others, in‐strength rates were highest in post‐single outburst contexts.

The two‐way ANOVA comparing ranked in‐strengths for dominance and conflict context was significant (F = 3.68, *df* = 7, 67, *p* = 0.002). Dominant females had higher in‐strength than other individuals (F = 6.35, *df* = 1, 67, *p* = 0.0141) and in‐strength differed between different conflict contexts (F = 5.43, *df* = 3, 67, *p* = 0.0021), with no significant interaction (F = 1.04, *df* = 3, 67, *p* = 0.3823). This indicates that dominant females received more proximity initiations than other individuals, and that rates of proximity initiation for all individuals varied between conflict contexts. In‐strength rates were highest in the hour following a single‐individual outburst for both dominant females and others (Figure 3).

**Figure 4:**
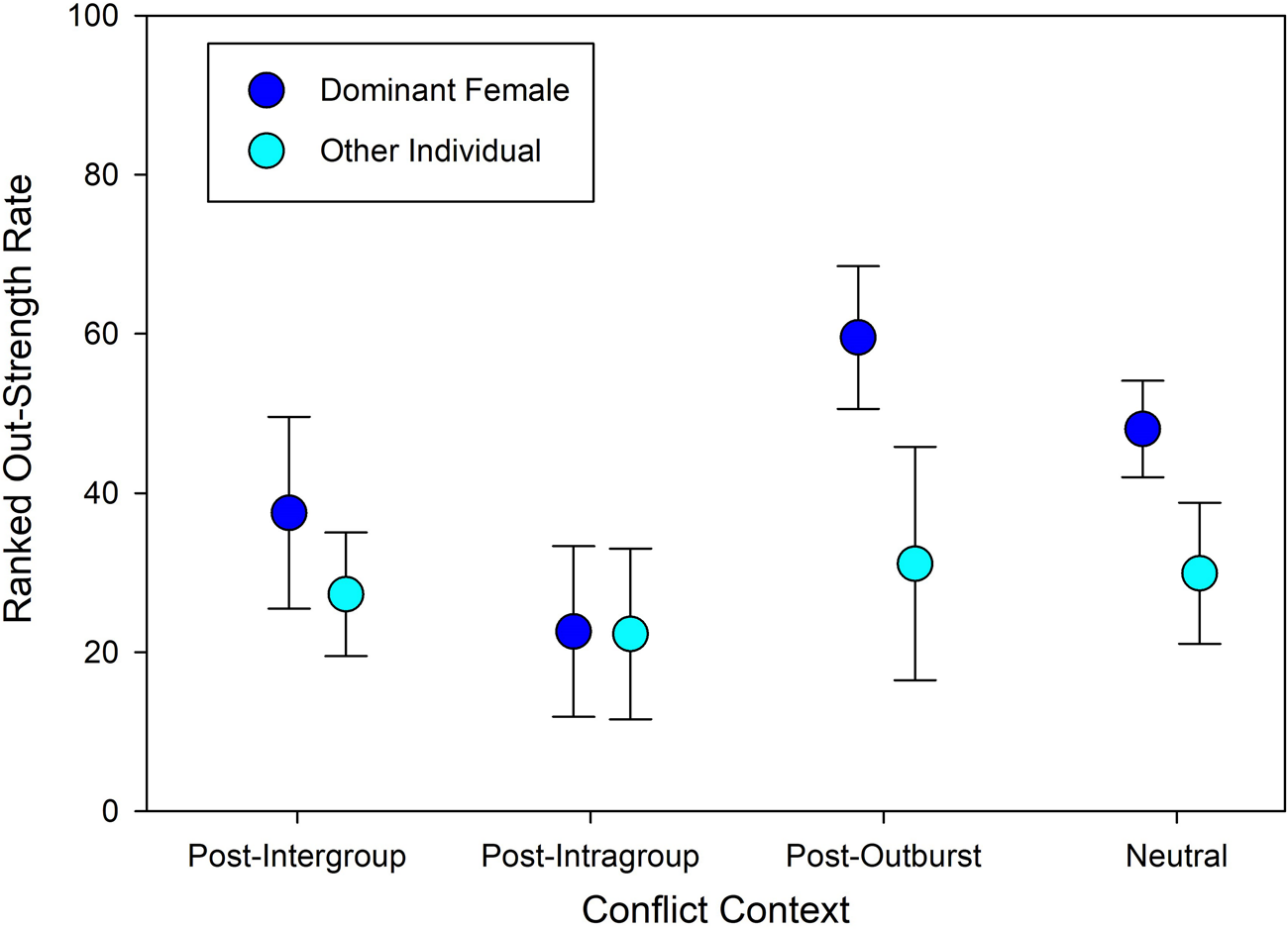
Ranked out‐strength rate comparisons between dominant females and other individuals across four conflict contexts with standard deviations: Out‐strength rate is measured as the rate of time spent in dyadic proximity bouts initiated by the subject. These rates are converted to ranks for a non‐parametric ANOVA. Out‐strength rates vary between conflict contexts, though dominant females did not have higher rates than other individuals. For both dominant females and others, in‐strength rates were highest in post‐single outburst contexts.

The two‐way ANOVA comparing ranked out‐strengths for dominance and conflict context was significant (F = 2.25, *df* = 7, 67, *p* = 0.0404). Dominant females did not have significantly higher out‐strength rates than others (F = 0.63, *df* = 1, 67, *p* = 0.43), though out‐strengths significantly varied by conflict context, showing a similar peak following single‐individual outbursts (F = 4.5, *df* = 3, 67, *p* = 0.0062). There was no significant interaction term (F = 0.55, *df* = 3, 67, *p* = 0.652). This indicates that dominant females and other individuals initiated proximity at similar rates, though these rates varied between conflict contexts (Figure 4).

### 3.3 Rates of Social Behaviors Across Conflict Contexts

Distribution of total strength scores was not normal for both dominant females (Kolmogorov‐Smirnov, D = 0.230753, *p* = 0.0222) and nondominant individuals (Kolmogorov‐Smirnov, D = 0.185009, *p* < 0.01), so total strength scores within both categories were converted to category‐specific ranks.

**Figure 5:**
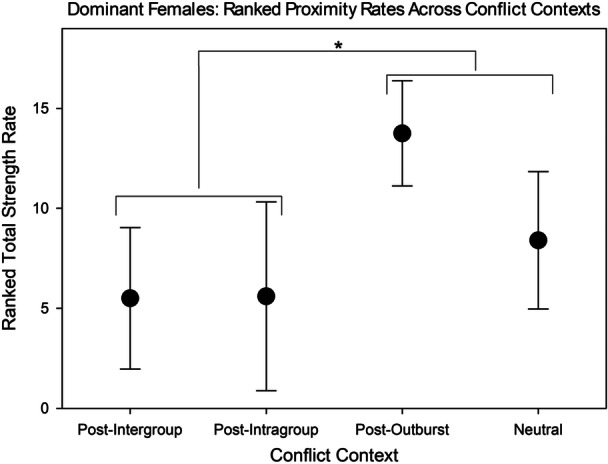
Comparisons of ranked proximity rates (or total strength rates) between conflict contexts for dominant females with standard deviations. Total strength rate is measured as the rate of time in which an individual is in dyadic proximity with another individual, regardless of who initiated. These rates are converted to ranks for a non‐parametric ANOVA. Ranked proximity rates were significantly lower post‐conflict (intragroup and intergroup) than in the neutral and post‐outburst contexts. Ranked proximity rates were higher post‐outburst than in the neutral context, though this difference was not significant.

For dominant females, the comparison of ranked overall rates of proximity across conflict contexts showed significant differences (F = 4.02, *df* = 3, 12, *p* = 0.0342). The multiple comparisons showed that non‐conflict contexts (neutral and post‐outburst) were significantly different from conflict contexts (post‐intergroup and post‐intragroup) (F = 7.51, *df* = 1, 12, *p* = 0.0179). Within non‐conflict contexts, it was highly suggestive that post‐outburst contexts had higher rates than neutral contexts, though this difference was not significant (F = 4.5, *df* = 1, 12, *p* = 0.0554). Within conflict contexts, post‐intragroup contexts were not different from post‐intragroup (F = 0, *df* = 1, 12, *p* = 0.9752). This indicates that dominant females engaged in social proximity more often following single‐individual outbursts and in neutral contexts than following intragroup or intergroup conflict, though this difference may have been driven by high proximity rates following outbursts and may not be as meaningful in regard to neutral contexts (Figure 5).

**Figure 6:**
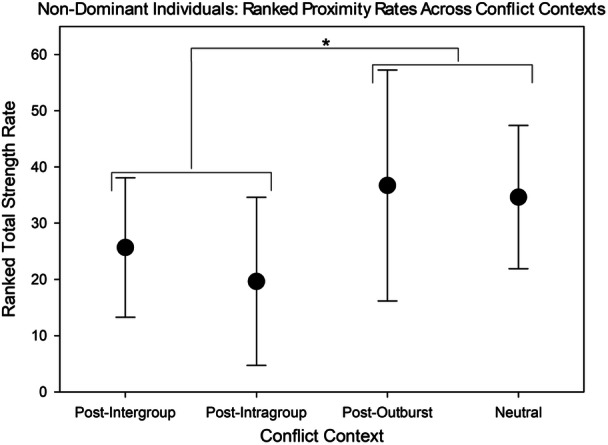
Comparisons of ranked proximity rates (or total strength rates) between conflict contexts for nondominant individuals with standard deviations. Total strength rate is measured as the rate of time in which an individual is in dyadic proximity with another individual, regardless of who initiated. These rates are converted to ranks for a non‐parametric ANOVA. Ranked proximity rates were significantly lower post‐conflict (intragroup and intergroup) than in the neutral and post‐outburst contexts.

For nondominant individuals, the comparison of ranked overall rates of proximity across conflict contexts showed significant differences (F = 4.07, *df* = 3, 55, *p* = 0.0111). The multiple comparisons showed that non‐conflict contexts (neutral and post‐outburst) were significantly different from conflict contexts (post‐intergroup and post‐intragroup) (F = 7.89, *df* = 1, 55, *p* = 0.0069). However, there were no differences within non‐conflict contexts (F = 0.15, *df* = 1, 55, *p* = 0.6985) or within conflict contexts (F = 0.63, *df* = 1, 55, *p* = 0.4303). This indicates that nondominant individuals engaged in social proximity more often following single‐individual outbursts and in neutral contexts than following intragroup or intergroup conflict (Figure 6).

### 3.4 Frequency of Sex Across Conflict Contexts

Overall frequency of sex post‐conflict (post‐intergroup and post‐intragroup) was significantly higher than expected given the amount of observation time for each context (Total G = 30.350, *df* = 5, *p* = 0.00001), with no significant heterogeneity in the results for each group. Of the six instances of post‐intragroup sex, four involved both individuals from the preceding conflict, and the remaining two involved one individual from the preceding conflict. All instances of sex in groups C and D were between groups, with an individual from each group copulating with each other through the mesh fence between them. All of these instances occurred following an intergroup conflict.

## 4 Discussion

This study demonstrates that dominant females are central to affiliative social networks, which aligns with prior research that emphasizes the integral role of dominant females. Dominant females also receive significantly higher rates of initiations from other group members, although they seem to initiate themselves at comparable rates to other group members. This indicates that the high centrality of dominant females is maintained through initiations from other group members. This does not mean that dominant female bonobos are passive in their social group, but that they do not maintain their social centrality through their initiations. In affiliative social states, they are likely attractive social partners and may exercise control in choosing the individuals with whom they accept/reciprocate affiliation.

Rates of initiations towards both dominant females and others were highest in the hour following a single‐individual outburst. Although overall proximity rates within dominant females were not significantly higher in single‐individual outburst states than in neutral states, we report a *p*‐value of 0.0554, which indicates that there may still be some difference between these states, particularly because we used a non‐parametric test, which is much less sensitive to small differences in data (Sokal and Rohlf 1987). These results are contrary to our predictions, in which we assumed that single‐individual outbursts would not be ‘enough’ of a disruption to the social group to result in behavioral changes from neutral contexts. Our results indicate that behavior within post‐outburst contexts is different from neutral contexts, yet also different from both post‐intragroup and post‐intergroup contexts. We believe that these outbursts may signal increased within‐group tension and possibly function as ‘pre‐conflict’ contexts: when group tension is high, and conflict is more likely to occur. In such a context, increased rates of proximity could function to counteract the increased tension through emotional/stress regulation or dyadic bonding and prepare oneself for a possible future conflict by affiliating with individuals that would be valuable as allies. These speculations require further investigation, though our results indicate that single‐individual outbursts may function differently from typical social conflict. Though rates of social state behaviors were significantly different when comparing neutral and post‐outburst states to post‐intergroup and post‐intragroup states, within dominant females, this difference is likely driven by the increase in social state behavior rates within post‐outburst states and should not be interpreted as a post‐conflict drop in sociality as compared to neutral states.

### 4.1 Limitations and Considerations

It is possible that idiosyncratic factors related to individual history may play a role in social network structure. Rodrigues and Boeving (2019) found that wild‐born bonobos exhibited greater eigenvector centrality and strength, and group tenure was significantly correlated with strength. Torfs et al. (2023) found higher eigenvector centrality and out‐strength among mother‐reared individuals versus atypically‐reared individuals. The population of bonobos in this study at the Milwaukee County Zoo was all born in captivity (Zookeeper communication, 2023), though it is still possible that early life experiences, zoo tenure, and personality differences may influence centrality and strength. Given that much of this information is unavailable to us, we are unable to conduct analyses for these factors but remain aware of their existence and possible influence.

It is also important to note that, due to these extensive possible influencing factors, results from single‐group studies should not be considered representative for an entire species (Stevens et al. 2008). This study involves five groups of unique compositions, though 6 of the total 18 bonobos appear in two groups, which means that groups A, B, and C (the groups with ‘repeat’ bonobos in their compositions) are not completely independent. Ideally, we would use ‘individual identity’ as a random factor in our analyses, but this is not possible because most bonobos in our data set do not have multiple centrality or strength scores per conflict context. However, bonobos are known to change their dominance status and behaviors based on group composition (Paoli et al. 2006; Caselli et al. 2023), so although certain elements such as early life history and personality (to an extent) follow the same individual between groups, we expect individuals to act differently (and be treated differently) between groups of different compositions. Therefore, we use this perspective to report and interpret our results, and we leave room for these results to be reinterpreted through individual‐level factors not taken into account. We encourage results reported here to be compared with similar studies for further validation and clarification.

Like many behavioral studies on captive apes, our study is limited in sample size. Although SNA has been conducted on primate groups as small as three or four individuals before (Kasper and Voelkl 2009; Létang et al. 2020; Torfs et al. 2023), researchers caution the use of indirect measures for connectedness like eigenvector centrality in small groups (Torfs et al. 2023 excluded groups of under five individuals for indirect SNA measures). Due to our limited access to larger groups, we include groups of three and four individuals in all analyses, though we acknowledge that broad biological interpretations may be limited.

As stated earlier, another limitation concerns our focal‐sampling methodology. It is possible that, should most reconciliations or reactions to conflict occur quickly after a conflict and not throughout the hour we consider ‘post‐conflict’, collecting behavioral data through 10‐min focal samples may miss important behavioral data from other individuals not being actively sampled. Focal sampling was used because we wanted to collect durations and initiations of social affiliation to create both nuanced and directed social networks. Further, the structure of the MCZ enclosures made it difficult to quickly find individuals for scan sampling. We would have liked to conduct simultaneous focal sampling on all individuals post‐conflict, but given that we had one observer (and at times, one research assistant) to collect all data, this was impossible. Because we only had one observer, we are also limited in ensuring reliability in behavioral observations.

## 5 Conclusion

The results of this study emphasize the flexible and adaptive nature of bonobo sociality, identify unique qualities of dominant female bonobo sociality, and highlight possible areas of future study. Captive bonobo groups change their behavior following instances of disruption and conflict, indicating that dyadic interactions can influence and change entire group dynamics and behaviors. Consistent differences in proximity and initiation rates were observed between dominant females and other group members, indicating that the social role of the dominant female in a bonobo social group is different from that of other bonobos and that this should be considered in future studies on bonobo social networks. Bonobo social intelligence, particularly as it applies to social cohesion, social roles, and peace‐making, may be expressed in more nuanced and complex ways than previously thought. Further, behaviors initially thought to be inconsequential during data collection (random single‐individual outbursts), showed significant results, and may signal important states of unrest that influence group‐level behavior.

This study is an example of how social network analysis can be used to assess both individual and group‐level behavioral change over time. Possible future directions include incorporating specific conflict information (individuals involved, resources present, outcome), individual histories, and genetic relationships, as well as using other behavioral markers of affiliation (space usage, affiliative/agonistic point behaviors, isolated affiliative behaviors). Further social network analyses are needed to validate our results and investigate possible functions of dominant female centrality, variations in in‐strength between both dominant female groupings and conflict states, and post‐outburst behavioral changes.

## Author Contributions

**Sedona Epstein:** conceptualization (lead), data curation (lead), formal analysis (lead), funding acquisition (lead), investigation (lead), methodology (lead), software (equal), supervision (equal), visualization (lead), writing – original draft (lead). **Mariam Fischer:** data curation (supporting), funding acquisition (supporting), investigation (supporting). **Sara Cotton:** investigation (supporting), project administration (supporting), writing – review and editing (supporting). **Frances White:** conceptualization (equal), data curation (equal), formal analysis (equal), funding acquisition (equal), methodology (equal), resources (lead), software (equal), supervision (equal), writing – review and editing (supporting).

## Supporting information

Supporting Information‐Ethogram Edits.

## Data Availability

Data is available on request from the authors.
