# Supplementary material for: Investigating Changes in Social Networks Following Conflict in Zoo‐Housed Bonobos (Pan paniscus)
Source: Am J Primatol. 2025 May 26;87(5):e70047. doi: 10.1002/ajp.70047 (PMC12105607; doi:10.1002/ajp.70047)
Supplement: Supplementary file 1 — Supporting Information‐Ethogram Edits. [file AJP-87-e70047-s001.docx]

| **Ethogram** | | |
| --- | --- | --- |
| **Category** | **Behavior** | **Definition** |
| Affiliative (Seconds) | Groom | Individual manipulates another individual’s body surface and hair with lips or fingers.  Initiator: Individual who moves towards or grabs dyadic partner to begin grooming or presents body part to dyadic partner to receive grooming.  Terminator: Individual who stops engaging in dyadic grooming first. They may stop grooming their dyadic partner or move away, or if they are being groomed, move away from individual grooming them. |
|  | Play | Individual engages in roughhousing, wrestling, chasing, or exaggerated movements or contact with another individual while displaying ‘play face’, relaxed and ‘loose’ body movements, or audible laughing sounds. Can be calm or rough.  Initiator: First individual to begin showing play behaviors. Often involves initiating ‘playful’ contact.  Terminator: First individual to stop showing play behaviors. May simply slow or stop, ignore dyadic partner, or begin another behavior. |
|  | Cofeed | Individual is within close proximity (1 meter) of another individual while one or both individuals are eating.  Initiator: Individual who initiates proximity when either of the dyad is eating.  Terminator: Individual who terminates proximity when either of the dyad is eating. |
|  | Contact | Individual is touching their body to another individual’s body.  Initiator: Individual who moves to begin initial contact.  Terminator: Individual who moves away to break contact. |
|  | Close Proximity | Individual is within 1 meter of another individual (typically one arm’s length away). Does not include if an individual is continuously passing by another individual without stopping or pausing.  Initiator: Individual moving towards dyadic recipient when 1 meter threshold is reached.  Terminator: Individual moving away from dyadic recipient when 1 meter threshold is broken. |
|  | Partial Proximity | Individual is within 2 meters of another individual (typically two arm’s lengths away). Does not include if an individual is continuously passing by another individual without stopping or pausing.  Initiator: Individual moving towards dyadic recipient when 2 meter threshold is reached.  Terminator: Individual moving away from dyadic recipient when 2 meter threshold is broken. |
| Agonistic (Count) | Displace | Individual approaches to within arm’s reach of another individual, or moves in direction of another individual with attention focused on them and the receiver retreats spatially or yields within 30 seconds  Social Modifier: Individual being displaced. |
|  | Pester | Individual repeatedly approaches another Individual, may throw things and/or swing above the receiver etc., is prepared to withdraw, and without pilo-erection or play face; at times resulting in full approaches or aimed throwing of objects without pilo-erection.  Social Modifier: Individual being pestered |
|  | Steal | Individual grabs food, object or infant from another individual and then runs away while carrying the food, object or infant.  Social Modifier: Individual from who the subject steals from. |
| Aggressive (Count, All Occurrence) | Aggressive Vocalization to Outgroup | Individual vocalizes in the direction of another group or to a specific member of another group. These include threat barks, loud hoots, and anger calls, and does not include ‘laugh’ vocalizations or food grunts/peeps. |
|  | Display | Individual runs tensed in the direction of, parallel to or closely passing by another individual, often while pushing an object. This can end in a collision or other contact. There is often a clear phase where the display is built up (body swaying)  Social Modifier: Individual who is the target of subject’s display. |
|  | Charge | Individual shows tensed running towards another individual.  Social Modifier: Individual the subject is charging towards. |
|  | Chase | Individual shows tensed running towards another individual while the other individual is running away. Chaser is actively adjusting route towards their target past the initial charge trajectory.  Social Modifier: Individual the subject is chasing. |
| Submissive (Count, All Occurrence) | Solicit Help | Individual stretches out arm and hand or turns head in the direction of another individual following or during an agonistic or aggressive interaction, often paired with a submissive grin.  Social Modifier: Individual from who subject is soliciting help. |
|  | Scream | Loud, high-pitched vocalization following or during an agonistic or aggressive interaction, often paired with a submissive grin or while fleeing from another individual.  Social Modifier: Individual from who |
|  | Flee | Individual moves away after a quick aggressive approach or charge without indication of play, and the fleeing lasts at least until the aggression stops  Social Modifier: Individual from who subject is fleeing from. |
| Single-Individual Outburst (Count, All Occurrence) | Scream, Hit Enclosure | Individual engages in an aggressive or submissive behavior with seemingly no trigger or recipient. This includes a ‘random’ scream or hitting of enclosure walls/glass. |
| Sociosexual (Count, All Occurrence) | Sex/Genito-Genital Rubbing | Copulatory or non-copulatory mount, often with thrusting, OR two female individuals rub genitalia together (can be in various positions). Does not include sex-inspect or massage with hands or mouth or masturbation.  Initiator: Individual may walk towards recipient, grab recipient, or show/present recipient their genitals to begin sexual contact. |
| Intervention (Count, All Occurrence) | Coalitionary | 3^rd^ party individual gets involved in a conflict between two other individuals and aggresses only one of them, effectively siding with the other. |
|  | Noncoalitionary | 3^rd^ party individual gets involved in a conflict between two other individuals and aggresses both of them, effectively not picking one side to support. |
| Table 3. Ethogram, based on Stevens et al. “Pictorial Bonobo Ethogram” | | |
